# Institutions, Parasites and the Persistence of In-group Preferences

**DOI:** 10.1371/journal.pone.0063642

**Published:** 2013-05-21

**Authors:** Daniel J. Hruschka, Joseph Henrich

**Affiliations:** 1 School of Human Evolution and Social Change, Arizona State University, Tempe, Arizona, United States of America; 2 Departments of Psychology and Economics, University of British Columbia, Vancouver, British Columbia, Canada; Durham University, United Kingdom

## Abstract

Much research has established reliable cross-population differences in motivations to invest in one’s in-group. We compare two current historical-evolutionary hypotheses for this variation based on (1) effective large-scale institutions and (2) pathogen threats by analyzing cross-national differences (N = 122) in in-group preferences measured in three ways. We find that the effectiveness of government institutions correlates with favoring in-group members, even when controlling for pathogen stress and world region, assessing reverse causality, and providing a check on endogeneity with an instrumental variable analysis. Conversely, pathogen stress shows inconsistent associations with in-group favoritism when controlling for government effectiveness. Moreover, pathogen stress shows little to no association with in-group favoritism within major world regions whereas government effectiveness does. These results suggest that variation in in-group preferences across contemporary nation-states is more consistent with a generalized response to institutions that meet basic needs rather than an evolved response dedicated to pathogens.

## Introduction

The degree to which people prefer interacting with and investing in family, friends, and in-group members–which we label “in-group preferences”–varies substantially across human societies, and has been associated with a variety of population-level cognitive differences [1]–[5]. For example, in one multi-country study of hypothetical decision-making, the probability of lying to help a friend over telling the truth in court varied between 5% and 70% [6]. Nevertheless, despite a large and expanding body of findings showing reliable differences across populations, only recently has research begun to develop and test historical-evolutionary causal explanations for such differences.

Here we assess two current historical-evolutionary accounts for this cross-cultural variation in in-group preferences, focused on the effects of (1) large-scale uncertainty-reducing institutions, and (2) pathogen threats.

The first *material or existential security* hypothesis proposes that these population-level differences are responses to the existence of social institutions that can buffer risk, ensure basic needs are met, and mitigate threats to survival [7]. Like other animals who engage in social niche construction, humans actively modify their social environments as a means of adapting to material threats, including pathogen stress [8], [9], environmental extremes [10], food insecurity [11], and inter-group conflict [12]. However, humans are unique in their ability to construct their social environments cumulatively over generations, with the cultural transmission of social norms (e.g., food sharing), knowledge (e.g., germ theory of disease), practices (e.g. food storage, charity), complex technologies (e.g., boiling water, burying the dead) and formal institutions (e.g., courts, police, hospitals, health care, insurance and social safety nets). In these culturally-constructed niches, humans face frequent decisions about investing in one’s family or in-group vs. pursuing other social investments, including cultivating new relationships in a broader social network. Under different social and ecological conditions, the same investments can have very different consequences. For example, public services, global markets, and social safety nets that mitigate material threats may render investments in an expansive network of kith and kin less necessary as alternative forms of social insurance. Moreover, limiting one′s social interactions to local in-group members can prevent one from accessing the benefits of trade and comparative advantage, of expanded mating opportunities, and of new ideas and cultural innovations. By contrast, in societies lacking such institutions, where plagues, injuries, and economic shocks represent serious and persistent threats, in-group members may be the only reliable source of social insurance and support, and intensive investments in enduring social relationships may serve as a crucial buffer against threats to survival and reproduction [13]–[15]. The cultural evolution of norms, know-how, technologies and institutions that increasingly mitigate threats to material insecurity may create new contexts which permit reallocations of investment away from in-group relationships via several mechanisms [15]–[17]. These can include facultative calculations of costs and benefits, learning over the lifespan, genetic changes, and culturally acquired beliefs, values, habits and motivations [10], [18], [19]. For example, a vast body of experimental work indicates that cuing uncertainty in a number of domains, including mortality, disease, and social exchange, makes people more likely to invest in cultivating cooperative social ties and to favor in-group members [20]–[25]. Conversely, priming individuals with terms related to safety and security make them less likely to favor in-group members [26]. This suggests that decisions about in-group and out-group investment involve at least some facultative responses to the current level of certainty and safety. These facultative responses and the other mechanisms outlined above may contribute to the extant patterns of variation in in-group investment. Some researchers have also proposed an opposite causal pathway linking in-group preferences and institutions. Specifically, lower levels of in-group favoritism may foster economic growth and the development of institutions that mitigate material threats [27]. In both cases, we would expect a correlation between institutional quality and in-group favoritism.

The second account proposes that in-group preferences are a form of behavioral immune system reflecting a cognitive adaptation evolved specifically to protect against the spread of pathogens. According to this hypothesis, in regions with high risk of infection by dangerous pathogens, individuals will preferentially affiliate with in-group members in a way that insulates them from infection by out-group members [8], [28]–[30]. Though originally predicting xenophobia (negative out-group attitudes and behaviors), the theory has been extended to account for in-group favoritism (positive in-group attitudes and behaviors) as well [30]. Depending on the specific treatment of this hypothesis, the adaptive mechanisms may range from short-term cost-benefit calculations to longer term changes due to cultural learning, epigenetics, or even genetic adaptation [28]. Emerging experimental evidence suggests that people do indeed adjust social motivations and behaviors (i.e. conformism) to specific cues of pathogen threats over and above generalized threats [31]. Broadly, this hypothesis is subsumed by the material insecurity hypothesis, which views pathogen threat as but one type of material insecurity. However, this hypothesis differs crucially from the material security hypothesis by positing that the adaptive mechanisms responsible for this effect are specific to pathogen risk and were designed to impede the spread of pathogens. In addition to critiques of the theory′s key assumptions [32], scholars have recently criticized cross-population tests of the pathogen stress hypothesis for not considering alternative hypotheses [31], [33] and for not accounting for the non-independence of country-level data [34].

Here we assess these two hypotheses using available cross-national measures of in-group preferences. We focus our analyses on three independent measures of in-group preferences used in the literature. First, we use Hofstede’s measure of collectivism as one of the first and most commonly deployed assessments of loyalty to one’s in-group in cross-national analyses. Second, Van der Vliert’s measure of in-group favoritism is a reliable between-country measure of in-group favoritism which incorporates in-group preferences at several social scales–including immediate family, extended relatives, and country. Third, Fincher and Thornhill’s measure of familism is a key variable in current studies of pathogen stress. We also further validate these findings against five additional measures of in-group favoritism–particularism, compatriotism, nepotism, familism, and embeddedness–in on-line File S1. These measures include preferences for in-groups of varying kinds and at differing social scales, from close friends and family to members of the same country.

To analyze these measures we used a three-pronged approach that goes beyond previous tests of the pathogen stress hypothesis. First, using ordinary least squares regression, we assess the effect of quality of basic government services (government effectiveness, GE) and parasite stress on all three assessments of in-group preferences, controlling for world region and dominant religious tradition. As a confirmatory check, we also look for evidence of reverse causality by which greater in-group favoritism might weaken large-scale institutions [27], [35], [36]. Specifically, we assess how our measures of in-group favoritism predict change in government effectiveness from 1996 to 2009. This approach further confirms that reverse causality is unlikely at least at relatively short 13-year time scales, though such reverse causality remains possible on larger time scales. Finally, we develop an instrumental variable regression as an additional check on selection and omitted variables in any observed relationship between government effectiveness and in-group preferences [37], [38].

Overall, these analyses suggest that general material insecurity in the face of weak institutions, not just a dedicated response to pathogens, is an important determinant of in-group preferences. Moreover, the instrumental variable analysis suggests a historical explanation for the raw, unadjusted correlations observed between pathogen stress and in-group favoritism.

## Materials and Methods

In this section we first discuss our sample and then how we measured preferences for in-group investment, institutional quality, pathogen risk, and religion. Then, we lay out the analysis and results.

### Sample

The units of analysis are geopolitical regions, which are usually formal countries (e.g. Italy), but also include regions defined by political, economic and cultural history (e.g. Hong Kong). Henceforth, we will refer to these units as “countries.” Countries can contain substantial within-population heterogeneity in cultural, religious and economic factors, but they also exhibit sufficient between-population variation to support informative ecological analyses [39]. The samples used in this paper differ depending on the availability of outcome measures, with sample sizes listed below.

### Measuring In-Group Preferences

For each variable, higher values indicate stronger in-group preferences. The derivation and description of these three in-group preference measures, five additional in-group preference measures, as well as predictor and control variables are described in Table S1, Table S2, and Table S3 in File S1.

#### Hofstede’s collectivism (N = 72)

Collectivism is the tendency to care about the consequences of one’s behavior for in-group members and to sacrifice personal interests for collective gains [2], [40]. The extreme individualism that distinguishes many western societies, by contrast, measures people’s lack of willingness to differentiate an in-group and sacrifice for the collective good of that in-group. We use Hofstede’s national measure of collectivism assessed from the work attitudes of over 100,000 IBM employees.

#### Van der Vliert’s in-Group favoritism (N = 121)

Van der Vliert [10] developed a scale of in-group favoritism from three highly correlated international assessments of: (1) familism, (2) nepotism, and (3) compatriotism (Cronbach’s α = 0.89). Familism is preferential concern for and investment in one’s closest relatives (parents, children and siblings) assessed from middle managers about how parents and children respect each other and live together [41], and this specific measure has also been used in other work as “in-group collectivism” [8]. Nepotism is favoring relatives over non-relatives in the allocation of resources, and was measured from a multi-country survey of business executives from nationally representative samples of firms about the degree to which senior management positions are chosen based either on superior qualifications or on one’s kin relationship [42]. Compatriotism is favoring members of one’s own nationality over others, and was derived from questions in the World Values Survey (1999–2002 wave) about whether employers should give priority to compatriots [43]. Additional analyses in the on-line file S1, confirm that the general results for this composite variable also hold for each of the three components.

#### Fincher and Thornhill’s strength of family ties (N = 71)

In order to compare our results with recent findings by Fincher and Thornhill about the pathogen stress hypothesis, we use the measure of in-group preference–strength of family ties–they use in a recent publication. Family investment is preferential concern for and investment in one’s closest relatives (parents, children and siblings). Fincher and Thornhill (2012) derived this measure as the sum of five items in the 1981–2007 pooled dataset of the World Values Survey about the value placed on immediate family. Despite capturing different dimensions of in-group preferences the three measures of in-group preference show moderate to high correlations among themselves (collectivism-favoritism ρ = 0.70, collectivism-family ties ρ = 0.65, favoritism-family ties ρ = 0.56, p<0.001).

### Measuring Government Services and Pathogen Stress

#### Pathogen stress

Estimates of contemporary pathogen prevalence were used from Fincher and Thornhill (2012). To assess F&T’s hypothesis about a dedicated psychological response to human-to-human pathogens, we focus on their preferred measure of non-zoonotic pathogens. In Table S4 in on-line file S1, we also assess the hypothesis with a historical measure of pathogen stress [9].

#### Quality of government services

To assess quality of government services, we used the World Bank’s 1996 measure of government effectiveness which indexes the quality of public and civil services in a country, including roads, schools, hospitals, and courts [44]. The on-line file S1 considers three other measures of institutions and material security: GDP per capita, the Human Development Index, and Food Stress (Table S5 in File S1).

#### Religion

To adjust for potential confounding effects of shared religious background [45], [46] [47], we use world religious tradition with a plurality of adherents in a country as determined by Inglehart and Norris (2004). The categories include Muslim, Jewish, Catholic, Orthodox, and Protestant, and Eastern (which includes Hindu, Buddhist, Shinto and Confucian traditions). We use Catholic as the reference category in regressions.

#### World region

To assess and adjust for potential confounding effects of shared social, political, and cultural history as well as shared genetic background, we use world regions defined by the World Bank, including sub-Saharan Africa, Middle East and North Africa, East Asia, South Asia, Latin America and the Caribbean, and Europe and Central Asia. We use Europe and Central Asia as the reference category in regressions. Such controls importantly assess whether observed associations could be due to unmeasured similarities among nation-states based on shared ecological, cultural, social or religious factors which are not causally related to key predictors. If observed associations don’t hold up under such controls, it is not possible to disentangle whether the effect of pathogens or institutions is due directly to these specific variables or rather to some underlying cultural or regional similarity which effects both pathogens or institutions and in-group favoritism. In short, including regional controls helps address the problem of the non-independence of countries as data points created by shared history, geography and proximity. Without such controls, Germany and Austria are considered as independent as Germany and Niger.

#### Additional control variables

We also assessed whether bivariate associations and model estimates changed when including a measure of income inequality in the models–the Gini coefficient measure closest to 1996 [48]. There were no substantive changes in effect sizes or inferences when including the Gini coefficient, and to maintain the largest sample size, we report results without Gini controls. We also assessed an interaction between government services and temperature variability based on prior analyses suggesting that this interaction may predict in-group favoritism [10].

#### Instrumental variable

Widely used in economics, an instrumental variable regression helps identify what part of the association between a predictor variable (X, government effectiveness in this case) and an outcome (Y, in-group favoritism in this case) is due to the direct effect of X on Y, rather than due to reverse causality of Y on X or from other omitted variables. An instrumental variable Z is a variable which is expected to cause the predictor variable (X), but whose effect on Y is mediated via X. An instrumental variable regression considers only the variation in X predicted by Z, and examines how this variation predicts the outcome Y. If a relationship between the variation in X predicted by the instrumental variable and the outcome can be shown, this contributes to establishing a causal relationship between X and Y more than a standard multiple regression. Following work in economics on historical determinants of economic growth [49], [50], we use the mortality rates of early settlers in European colonies (1600–1875) as an instrumental variable which is expected to affect contemporary government effectiveness. Acemoglu et al. provide ample historical evidence that European colonizers avoided settling in places with high mortality rates, such as in the Belgian Congo. In lieu of settling, they set up extractive systems in these places. In situations of low mortality, on the other hand, colonizers settled in larger numbers and brought with them institutions, such as respect of private property, checks and balances in government, and equality of opportunity, which in turn fostered greater government effectiveness that persisted even after independence [49]. These measures of settler mortality allow us to identify what portion of the variance in government institutions is due to early (exogenously caused) settlement patterns. Given this reasoning and the strong association between early settler mortality and contemporary government effectiveness (ρ = −0.54, N = 55), we use settler mortality (1600–1875, [49], [51]) as an instrumental variable for the relationship between effectiveness of government institutions and in-group preferences. More details on this approach are provided in the online file S1.

## Results

Here we present analyses of three measures of in-group preferences using one measure of public services and one measure of pathogen stress. Additional analyses of other measures of in-group preferences, pathogen stress, and material security as well as tests of potential interactions are presented in the on-line file S1.

**Figure 1:**
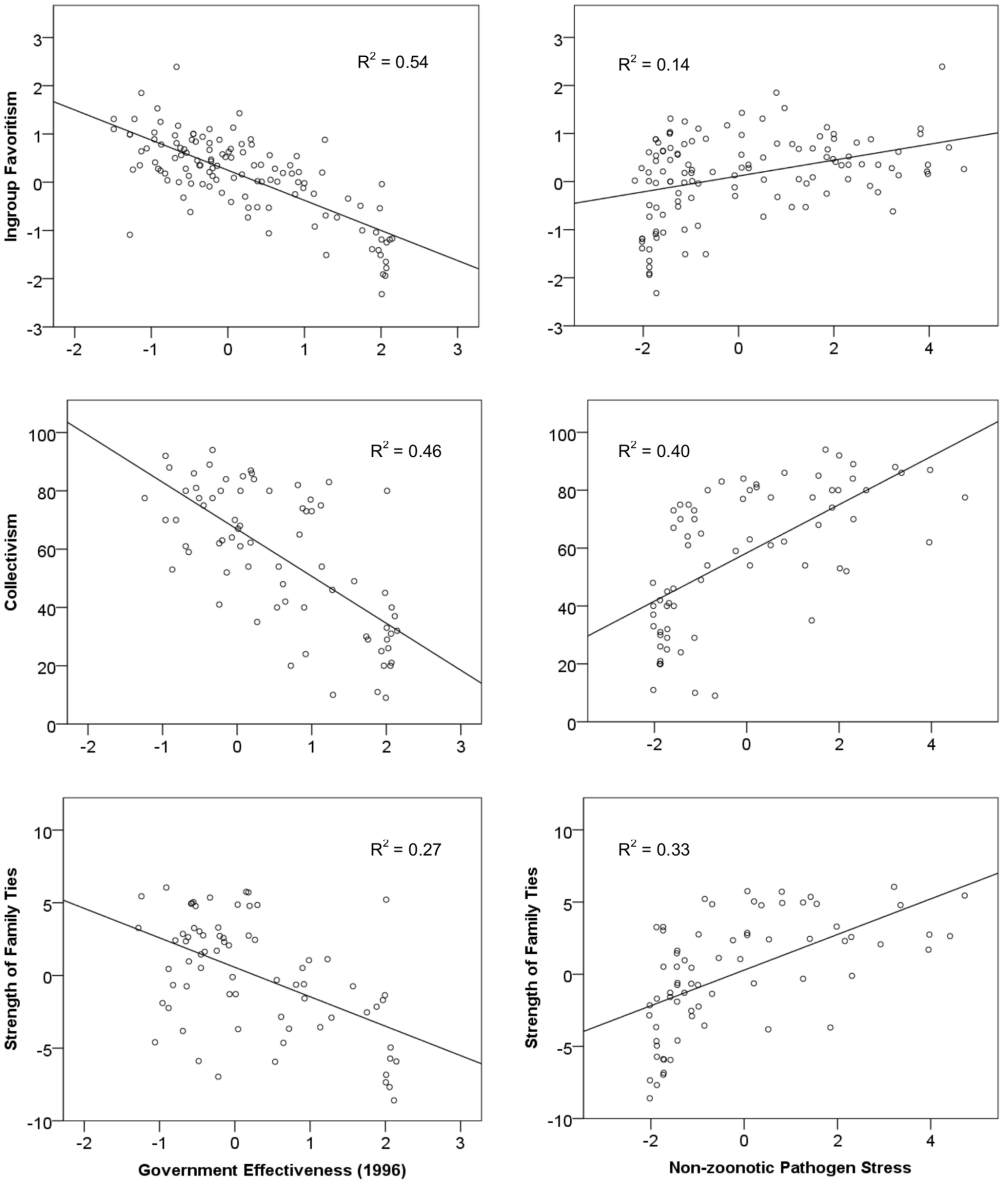
Three Measures of In-group Preferences by Government Effectiveness and Pathogen Stress.

In bivariate correlations with in-group preferences, both government effectiveness (ρ = −0.52, −0.68, −0.74, p<0.001) and pathogen stress (ρ = 0.58, 0.64, 0.37, p<0.001) were significantly associated with all three measures of in-group preferences–strength of family ties, collectivism, and in-group favoritism, respectively (Figure 1). When including government effectiveness and non-zoonotic pathogen stress together in a linear regression predicting in-group favoritism, government effectiveness remained significantly associated with all three primary measures (and all five alternative measures) of in-group favoritism. In the regression, pathogen stress showed less consistent associations with in-group favoritism measures. It was significantly associated with only two of the three primary variables–Collectivism and Strength of Family ties–and only two of the five alternative measures–Nepotism and Embeddedness.

**Figure 2:**
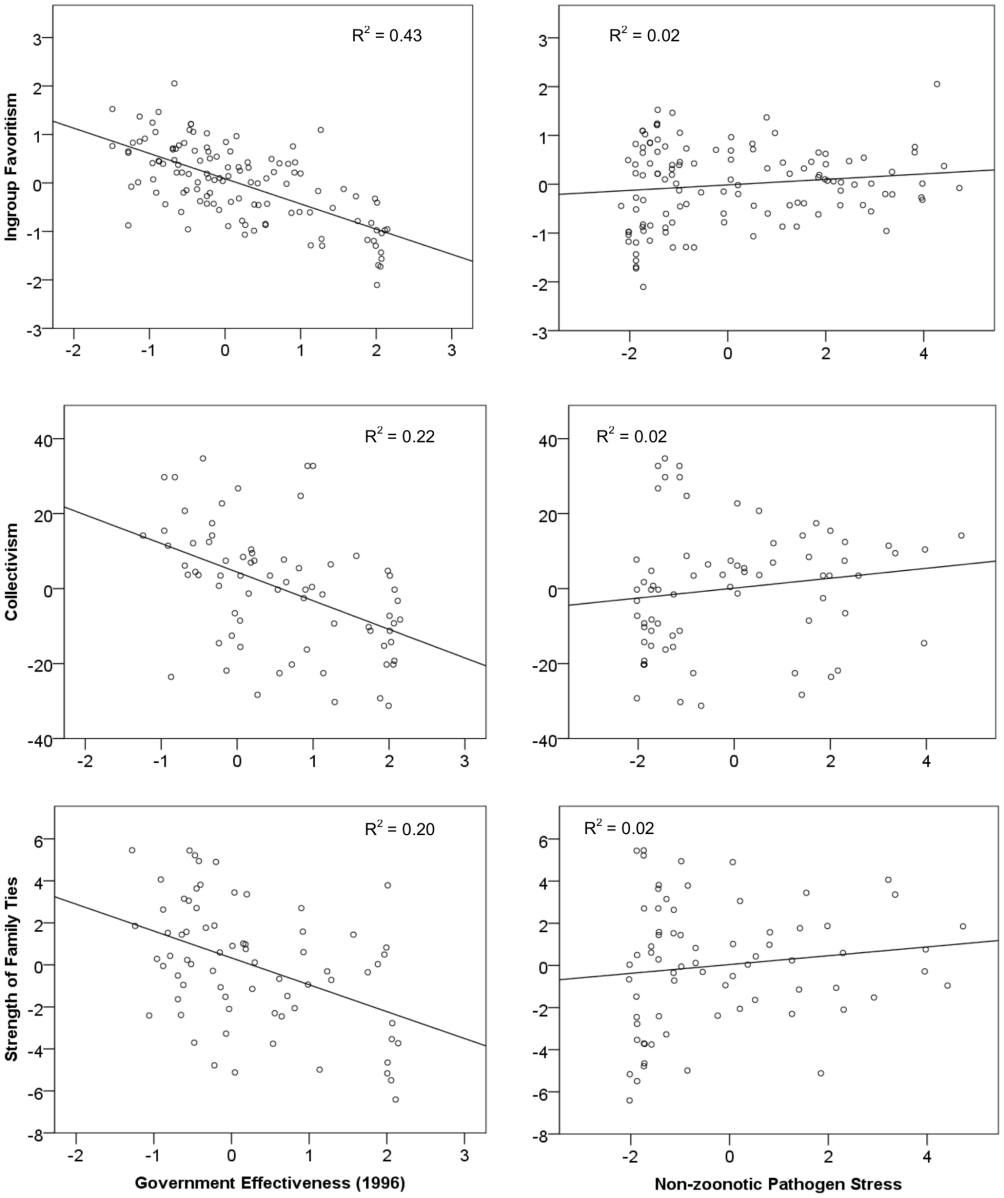
Three Measures of In-group Preferences (residualized by world region) by Government Effectiveness and Pathogen Stress.

We fit regression models of each of the three measures of in-group preferences on Government Effectiveness (GE) and Pathogen Stress (PS) controlling for (1) world region alone and (2) both world region and dominant religion. The standardized regression coefficients in Table 1 show that after controlling for shared regional background, GE is significantly related to Collectivism (standardized beta = −0.54, ΔR^2^ when adding Collectivism to regional model = 0.17), Ingroup Favoritism (standardize beta = −0.75, ΔR^2^ = 0.48), and Strength of Family Ties (standardized beta = −0.36, ΔR^2^ = 0.13). This is consistent with the five other measures of in-group measures in the on-line file S1. No associations between in-group preference measures and non-zoonotic pathogen prevalence remained significant after controlling for world region. In the on-line file S1, we show that historical pathogen stress remains associated with one of the three primary outcomes–Strength of Family Ties, p = 0.015–but not with any of the other five variables included in the on-line file S1. Figure 2 graphically shows the relationship of the in-group preference measures with GE and PS, when the impact of world region has been removed. Within-region analyses of the association of government effectiveness and pathogen stress with in-group favoritism measures are consistent with these findings (see on-line file S1).

**Table 1:** Regression models predicting 3 measures of in-group preference by government effectiveness, pathogen stress, and dominant religion (Coefficients are standardized betas).

	Collectivism			Ingroup Favoritism			Strength of Family Ties		
	N = 72			N = 121			N = 71		
	1	2	3	1	2	3	1	2	3
Government Effectiveness (GE)	−0.47***	−0.52***	−.031*	−0.72***	−0.75***	−0.63***	−0.35*	−0.36***	−.08
Pathogen Stress (PS)	0.34*	−0.01	−0.06	0.03	0.05	−0.01	0.45***	0.14	0.13
**Religion**									
Catholic			–			–			–
Protestant			−0.27**			−0.33***			−0.33*
Orthodox			0.14			0.05			0.08
Islam			0.09			−0.08			0.30*
Eastern			−0.03			−0.08			−0.08
Jewish			0.02			−0.09			–
Adjusted R^2^	0.52	0.66	0.72	0.53	0.57	0.64	0.43	0.53	0.63
ΔR^2^ from adding GE & PS	–	0.17	0.03	–	0.48	0.22	–	0.13	0.00

* p<0.05,

** p<0.005,

*** p<0.001.

Models 2 and 3 include regional controls. ΔR^2^ is the increase in adjusted R^2^ when adding Government Effectiveness and Pathogen Stress Base Model.

After adding controls for dominant religion, GE remains significantly related to Collectivism (ΔR^2^ when adding GE to region+religion model = 0.03) and Ingroup Favoritism (ΔR^2^ = 0.22), which is consistent with four other measures of in-group measures modeled in on-line file S1, but not with Strength of Family Ties. In all cases, adding dominant religion to the model significantly reduces the independent variation accounted for by GE. Importantly, collinearity statistics indicated no substantial problems with collinearity in these models (all tolerances >0.20 and VIF<5).

In the full model including region, religion, government effectiveness and parasite stress, a country’s predominant religion accounted for additional variation in in-group preferences across all measures of in-group preferences. Table 1 shows that, adjusting for GE and PS variables, Protestant religion most consistently affected in-group preferences. Countries with a plurality of Protestants had lower average in-group preferences for all three measures. In the full model, regions show less consistent relationships with in-group favoritism, with East Asian and sub-Saharan African countries showing significantly higher levels of collectivism and strength of family ties, Latin American countries show significantly higher levels of collectivism, and North African and Middle Eastern countries show higher levels of in-group favoritism (Tables S8 & S9 in File S1).

To assess whether the observed associations between the effectiveness of government institutions and in-group preferences are due to confounding or omitted variables, we conducted two checks (full analyses available in on-line file S1).

For the first check, we estimated how well in-group preferences predicted change*s* in GE from 1996 to 2009 as well as related measures of GDP per capita from 1996 to 2009 and the UN Human Development Index from 1995 to 2010, adjusting for geographic region and dominant religion. The effects were either non-significant or significant in the opposite direction expected by an argument for reverse causality (Table S6 in File S1). Thus the cross-sectional association between in-group preferences and these measures is unlikely a result of in-group preferences leading to higher levels of material insecurity or depressed economic growth at least at a 13-year time scale. If anything, the opposite is true.

The second check involved an instrumental variables regression and followed Acemoglu et al. [49] by using settler mortality during colonization as an exogenous source of variation in later quality of government institutions. We find that the estimates from the original OLS regression are consistent with the estimates from the instrumental variable regression, indicating that omitted variables have not introduced substantial bias (Table S7 and Figures S1–S3 in File S1). In fact, for all three of our measures of in-group favoritism, the IV coefficient estimates are larger in magnitude than the OLS coefficients, and for Collectivism, they are significantly larger in magnitude. This suggests that any endogeneity issues we have not modeled–if anything–likely suppress the size of the observed relationship.

These findings are robust to a variety of checks and alternative hypotheses. Tables in the on-line file S1 provide analyses parallel to those shown above for all eight of the available measures of in-group preferences, including individual analyses of the measures that compose Van der Vliert’s In-Group Favoritism (Tables S8 & S9 in File S1) and various measures of pathogen stress, including both historical pathogen stress and zoonotic pathogen stress (Table S4 in File S1). Tables S10 to S12 in File S1 show that including an interaction term for GE and PS does not improve the model, that including a term for Temperature Range and the interaction of Temperature Range and GE does not improve the model, and that historical pathogens do not confound the relationship between GE and In-group Favoritism.

## Discussion

Cross-national variation in in-group preferences or favoritism, measured in three distinct ways, reveal a consistent relationship between government effectiveness and in-group preferences. Specifically, in societies where government services are less likely to meet people’s basic needs, people invest preferentially in family and in-group members. This finding remains for all three of our in-group preferences measures when both pathogen stress and world region are included in the analysis. The effect is robust across alternative proxies for government effectiveness as well as all five alternative measures of in-group favoritism considered in the on-line file S1. These effects also remain for two of three measures (and four of five supplementary measures) even after removing global level variation in religious denomination. Finally, these effects withstand checks on reverse causality and omitted confounding and selection.

Contrary to a recent finding that specific psychological responses to pathogens explain this cross-population variation [8], there is no significant effect of non-zoonotic pathogen stress on any of the three measures of in-group preferences (or the five supplementary measures) after including controls for geography and shared cultural history. Even when simply controlling for government effectiveness, parasite stress only remains significantly associated with four of the eight measures of in-group preferences. Moreover, when these associations are significant, the coefficients on pathogen stress predictor variables are no larger than other material security measures. These findings indicate that pathogens are inconsistently associated with measures of in-group favoritism when controlling for government effectiveness, that significant associations may be due to confounding from other variables which covary across major world regions, and that the effects of pathogens are generally weaker than the effects of government institutions. Taken together, these findings suggest that a generalized response to social resources available to meet basic needs (which may include buffers against disease threats) appears the more plausible adaptive account for variation in in-group preferences, than a response dedicated specifically to pathogens.

We also identified an independent contribution of shared religious heritage to in-group preferences that accounted for a substantial portion of the effect of institutions on in-group preferences. A large part of this effect is carried by Protestantism, and countries with a plurality of Protestant adherents have significantly lower levels of in-group favoritism even after controlling for government effectiveness and world region. This is consistent with Weber’s view that a key effect of Protestantism was to “shatter the fetters” of extended family, and presumably other kinds of in-groups [52]. Recent authors have pinned this on Protestant core values of self-reliance and individualism which potentially led to less investment in family, friends and local in-groups [53], [54]. The current data is not equipped to discriminate between hypotheses for the role that dominant religion plays in the relationship between institutions and in-group preferences, though these findings are consistent with work suggesting that modern religions have evolved culturally to expand the sphere of social interaction, cooperation and exchange [55]. Taken together, these findings suggest that both general processes of adaptation to material insecurity, as well as particular historical contingencies or trajectories, may play a role in shaping people’s in-group preferences.

We argue that variation in institutional resources creates the relevant social niche to which a variety of in-group preferences may be a response. However humans also possess several different mechanisms that permit adaptation at different time scales, including immediate cost-benefit calculations, learning over the life course, and cross-generational transmission [56]–[61]. The current data is insufficient to discriminate between these different pathways [28]. Some researchers have proposed that high endemic pathogen load accounts for the observed link between low institutional quality and in-group preferences by both: (1) inhibiting economic growth and the development of public services [62] and (2) spurring in-group favoritism. A related argument proposes that high pathogen load leads to in-group preferences, which in turn lead to weak institutions [63]. Two of our findings suggest these proposals are unlikely. First, the effect of pathogen prevalence on in-group favoritism generally does not withstand simple controls for common regional and religious background. This suggests that the second pathway is not well-supported by existing cross-national data. Second, the effects of other measures of material security on in-group preferences are usually stronger than are pathogen stress, indicating that pathogen stress is not a relevant confounder. A more plausible explanation based on our analysis would place the causal role of pathogens (at least among former European colonies) at much deeper time scales [50]. Specifically, places with low pathogen stress led European colonizers to settle and to forge effective institutions. In places with high pathogen stress, colonizers set up extractive regimes with little concern for fostering effective institutions. That is, pathogen stress may have influenced the spread of effective, pluralistic, government institutions, which in turn influences in-group favoritism (and GDP per capita). Consistent with this hypothesis, government effectiveness significantly mediates the effect of historical pathogens on seven of the eight measures of contemporary in-group preferences (see on-line file S1). However, future studies that go beyond cross-sectional, cross-national datasets will be necessary to disentangle such potential interactions.

There are a number of limitations to our cross-sectional, cross-national analyses. We are analyzing aggregate decisions based on aggregate predictors, and it is possible that the associations do not reflect between-individual differences in decisions and adaptations–though other work suggests they do [58]. Second, since most data was only available at single time points, it is difficult to sort out the causal direction underlying observed associations. That said, when longitudinal data was available, we have tried to assess the possibility of reverse causation. Checks on reverse causation suggest that greater in-group preferences at the national level are not associated with reductions in government effectiveness over a 13-year period. Thus, there is little support for the claim that the cross-sectional association results from in-group preferences decreasing government effectiveness at least over the short run. Third, our controls for shared culture–World Bank region and dominant world religion–are admittedly coarse-grained. However, they do help discriminate between the government effectiveness and parasite stress hypotheses. Future work, will hopefully apply more sophisticated checks on Galton’s problem or at least determine that they are unnecessary. Fourth, the national level measures of in-group favoritism we use in this study tap into only some aspects of in-group favoritism and are available for limited samples of countries. Future analyses with measures that cover a more representative sample of countries and examine in-group favoritism at differing social scales and in different social situations will provide important refinements of these analyses. Finally, with observational studies there is always the problem of unmeasured confounding. An instrumental variable analysis indicates that the results are robust to omitted confounding or selection. We have also examined two omnibus sources of confounding, world region and dominant religious traditions, as well as economic inequality. The first two capture geography, shared cultural history and other effects, such as those associated with colonization and religious assimilation. If alternative theories are proposed, it may be possible to identify variables for assessing such confounding.

Here we have focused on only one kind of cultural niche construction, how institutions, pathogens, economic growth and technologies have shaped a variety of cultural and behavioral responses toward in-group members. Humans also devote considerable time and effort to investing in religious activities, such as attending religious services and praying, which can be framed as an investment in relationships with supernatural entities. Interestingly, cross-national studies of religious investment–e.g., praying and attending services–indicate similar associations with material security [43]. Strong secular institutions may create cultural evolutionary pressures for different forms of religiosity or spirituality. Future work that examines the influence of material security on this and other kinds of social niche construction will hopefully shed light on the nature and bounds of this association.

## Supporting Information

File S1 The file “S1” contains supporting information on study measures and additional analyses.
(DOC)
