# Supplementary material for: Institutions, Parasites and the Persistence of In-group Preferences
Source: PLoS One. 2013 May 21;8(5):e63642. doi: 10.1371/journal.pone.0063642 (PMC3660589; doi:10.1371/journal.pone.0063642)
Supplement: File S1 — The file “S1” contains supporting information on study measures and additional analyses. (DOC) [file pone.0063642.s001.doc]

# Derivation and description of variables

## In-group Preferences

Our eight measures of in-group preferences vary in terms of their emphasis on: (1) kin relationships (family members and friends and compatriots), (2) size of in-group (friends and family vs. compatriots), (3) the nature of observation (reports of others’ behaviors vs. report of own behaviors and preferences), and (4) the social tradeoff involved (investment in self, in out-group members, in following a norm, or no specific tradeoff). Here we describe the source and derivation of these variables.

### The three measures used in the Main text

*Van der Vliert’s In-Group Favoritism.* Van der Vliert developed a scale of in-group favoritism from three highly correlated international assessments of: (1) familism, (2) nepotism, and (3) compatriotism (Cronbach’s α = 0.89). Familism is preferential concern for and investment in one’s closest relatives (parents, children and siblings) and is assessed from middle managers in each of 60 countries about how parents and children respect each other and live together . Nepotism is favoring relatives over non-relatives in the allocation of resources, and was measured from a multi-country survey of business executives from nationally representative samples of firms about the degree to which senior management positions are chosen based either on superior qualifications or on one’s kin relationship . Compatriotism is favoring members of the one’s own nationality over others, and was derived from questions in the World Values Survey (1999-2002 wave) about whether employers should give priority to compatriots . Further explanation of these scale components is provided under “Alternative Measures” below.

*Collectivism.* Collectivism is the tendency to care about the consequences of one’s behavior for in-group members and to be willing to sacrifice personal interests for collective gains . One of the most commonly used measures of collectivism at the national level is reported by Hofstede (2001) who assessed work attitudes from over 100,000 IBM employees worldwide. From these data Hofstede estimated collectivism scores for 68 specific geopolitical regions included in our analyses. Hofstede’s measure correlates strongly with an alternative measure constructed by Suh et al. (ρ = 0.91), and Suh’s score was used to calculate a comparable collectivism score for Nepal, Nigeria, Zimbabwe, and Egypt which were not reported in Hofstede (Hofstede = -9.537+Suh*10.749). We use an inverse of Hofstede’s score (100 – individualism), so that a higher score indicates greater collectivism.

*Fincher and Thornhill’s Strength of Family Ties.* In order to compare our results with recent findings by Fincher and Thornhill about the pathogen stress hypothesis, we use the measure of in-group preference—strength of family ties—they use in a recent publication. Fincher and Thornhill (2012) derived this measure as the sum of five items in the 1981-2007 pooled dataset of the World Values Survey about the value placed on immediate family. The items included statements about the importance of: (1) family in one’s life, (2) loving and respecting parents despite their faults, (3) doing one’s best for one’s children even at expense of one’s own well-being, and (4) making one’s parents proud. The last item asked if people lived with their parents (Cronbach’s α = 0.86).

### Five Alternative measures

*Familism.* Family investment is preferential investment in one’s closest relatives (parents, children and siblings). This is the first component of Van der Vliert’s measure of ingroup favoritism (see above). This measure is based on data from House et al. (2004) and derived by Van de Vliert (2011). Between 1994 and 1997, House et al. sampled middle managers (*n* = 17,370) from domestic organizations in each of 60 countries in the target industries of food processing, financial services, and telecommunications . Participants answered four questions about interactions with family members as observed in their society (1 = *strongly disagree*, 7 = *strongly agree*). These were: “In this society, children take pride in the individual accomplishments of their parents”, “In this society, parents take pride in the individual accomplishments of their children”, “In this society, aging parents generally live at home with their children”, “In this society, children generally live at home with their parents until they get married”. The internal consistency of these four items was good (Cronbach’s α = .77), and reported estimates are response bias corrected . A higher score indicates greater familism.

*Nepotism.* Nepotism is the favoring of relatives over non-relatives in the allocation of resources. This is the second component of Van der Vliert’s measure of ingroup favoritism (see above). Data were used from a multi-country survey of business executives from nationally representative samples of firms . Executives responded to a 7-point likert scale, “Senior management positions in your country are: (1) held by professional managers chosen based on superior qualifications, …, (7) usually held by relatives.” Here we use standardized values provided by Van de Vliert (2011).

*Compatriotism.* Compatriotism is favoring members of the one’s own nationality over others. This is the third component of Van der Vliert’s measure of ingroup favoritism (see above), and is derived from data for nationally representative subsamples of adults from 73 countries from the 1999-2002 wave of the World Values Survey . Professional interviewers substituted their own nationality for “British” when asking: “Do you agree or not agree with the following statement? When jobs are scarce, employers should give priority to [British] people over immigrants” (3-point response scale: agree, disagree, or neither). We use the variable reported by Van de Vliert (2011), which is a standardized score based on the percentage of individuals in a country who agreed with the statement.

*Schwartz’s Cultural Embeddedness.* Based on a reviewer’s earlier suggestion, we included Schwartz’s dimension of cultural embeddedness as an alternative measure of in-group preference. This dimension captures the relationship between the individual and the group, and involves an emphasis on maintenance of the status quo, propriety, and restraint of actions or inclinations that might disrupt group solidarity or the traditional order .

*Particularism*. Particularism is the preference for helping kith and kin over following universally applicable rules of fairness . In several publications, Trompenaars and Hampden-Turner describe the responses of multi-national corporate managers to the passenger’s dilemma, whereby one must make the choice between telling the truth under oath and helping a friend . The variable reported by Trompenaars and Hampden-Turner is the probability that a respondent from a country stated either: (1) that the friend has a definite right to expect the respondent to lie for him or (2) that the respondent would lie under oath. As data was updated in successive publications, more recent publications take precedence over earlier publications. This measure of particularism correlates moderately with other measures collected by Trompenaars and Hampden, including the willingness to give a friend insider information from one’s corporation (ρ = 0.66, 1993, *n* =35 countries), the willingness to lie about a friend’s medical exam to improve his insurance premium (ρ =0.51, 1998, *n* = 28), and the willingness to lie about a friend’s restaurant in a published review (ρ = 0.65,0.69, 1993,1998, *n* = 32, *n* = 30).

## Institutions and Material security

The key variable used for our analyses in the main text was the World Bank measure of government effectiveness as a measure of quality of government services. In the supplementary materials below we also examine related measures that assess: (1) material resources available per capita (GDP per capita), (2) general material security, and (3) food security.

*Quality of Government Services (1996):* To assess quality of government services, we used the World Bank’s measure of government effectiveness which indexes the quality of public and civil services in a country, including roads, schools, hospitals, and courts[[1]](#endnote-2).

*Material Resources:* To assess material resources, we used log(GDP per capita) (World Bank 1996 measure, gross domestic product per capita purchasing power parity in 2005 dollars).

*Material Security:* We used the United Nations human development index (HDI), which Norris and Inglehart have used previously as a measure of “existential security” grounded in social and economic development . This measure includes indices of health, and thus is directly measuring both the quality of institutions and pathogen stress. For this reason, we do not use this as the primary measure for analyses.

*Food Insecurity:* Food insecurity was assessed using country-level data for proportion of total household consumption expenditure devoted to food.[[2]](#endnote-3) The larger share of overall household consumption devoted to food, the more sensitive household budgets are to changing food prices and the more prone they are too food insecurity.

## Predominant Religion

We use world religious tradition with a plurality of adherents in a country as determined by Inglehart and Norris (2004). The categories included Muslim, Jewish, Christian-Catholic, Christian-Orthodox, and Christian-Protestant, and Eastern (which included a combination of Hindu, Buddhist, Shinto and Confucian traditions). Eastern religions were aggregated into a single category as no single religion was sufficiently numerous in the sample to permit further stratification. When Inglehart and Norris did not specify the world religion, we assessed the world religion having a plurality of adherents in the country . We use Catholic as the reference category in regressions.

## World Region

To adjust for potential confounding effects of shared social, political, and cultural history, we use world regions defined by the World Bank, including sub-Saharan Africa, Middle East and North Africa, East Asia, South Asia, Latin America and the Caribbean, and Europe and Central Asia. The World Bank does not classify upper income countries. We classified upper income countries based on shared cultural heritage--Japan as East Asia, continental European countries, U.K., U.S., Canada, New Zealand and Australia under Europe/Central Asia. We use Europe and Central Asia as the reference category in regressions.

## Description of Key Quantitative VAriables

| **Table S1. Descriptives for key variables** | | |
| --- | --- | --- |
| **Variables** | **N** | **Mean (SD)** |
| **Key Ingroup Measures** |  |  |
| Van der Vliert Ingroup Favoritism | 121 | 0.14 (0.86) |
| Hofstede Collectivism | 72 | 57.5 (23.7) |
| F & T Strength of Family Ties | 71 | 0.03 (3.91) |
| **Alternative Ingroup Measures** |  |  |
| Schwartz Cultural Embeddedness | 71 | 10.3 (5.3) |
| Familism | 57 | 0.00 (1.00) |
| Nepotism | 118 | 0.01 (1.00) |
| Compatriotism | 76 | 0.01 (1.00) |
| Particularism | 43 | 27.9 (17.6) |
|  |  |  |
| **Institutions and Material Security** |  |  |
| Quality of Public Services | 128 | 0.16 (1.00) |
| Human Development Index | 122 | 0.72 (0.20) |
| GDP per capita (PPP in 1000 USD) | 123 | 10.89 (11.13) |
| % of household budget on food | 113 | 0.40 (0.17) |
|  |  |  |
| **Pathogen Stress** |  |  |
| Non-Zoonotic Pathogen Prevalence | 128 | 0.14 (1.93) |
| Zoonotic Pathogen Prevalence | 128 | 0.46 (0.88) |
| Historical pathogen prevalence | 128 | 0.08 (0.63) |

| **Table S2. Bivariate correlations between institutions, material security, and pathogen stress measures.** Sample sizes in parentheses. GE = government effectiveness. | | | | | | |
| --- | --- | --- | --- | --- | --- | --- |
| **Variable** | **GE** | **Log(GDP)** | **HDI** | **FS** | **NZPS** | **ZPS** |
| Log(GDP) | 0.78  (123) |  |  |  |  |  |
| Human Development Index  (HDI) | 0.70  (122) | 0.93  (119) |  |  |  |  |
| Food Insecurity (FS) | -0.75 (112) | -0.76 (109) | -0.70 (107) |  |  |  |
| Non-zoonotic Pathogen Stress  (NZPS) | -0.50 (128) | -0.64 (123) | -0.66 (122) | 0.28** (112) |  |  |
| Zoonotic Pathogen Stress (ZPS) | -0.19 (128)* | -0.22 (123)* | **-0.16 (122)** | **0.06**  **(112)** | 0.52  (128) |  |
| Historical Pathogen Prevalence (HPP) | -0.54 (127) | -0.63 (122) | -0.62 (122) | 0.37  (112) | 0.79 (127***)*** | 0.36  (127) |
| Significant at 0.001 level unless otherwise noted. * p < 0.05, ** p < 0.005. *Non-significant* results (> 0.05 level) are in bold italics. | | | | | | |

| **Table S3. Bivariate correlations between in-group preferences.** Sample sizes in parentheses. | | | | | | | |
| --- | --- | --- | --- | --- | --- | --- | --- |
| **Variable** | **Collect** | **IN** | **FA** | **NP** | **CO** | **PA** | **EM** |
| Ingroup (IN) | 0.70 (70) |  |  |  |  |  |  |
| Familism (FA) | 0.74 (49) | 0.93 (57) |  |  |  |  |  |
| Nepotism (NP) | 0.66 (69) | 0.87 (118) | 0.69 (56) |  |  |  |  |
| Compatriotism (CO) | 0.59 (58) | 0.85 (76) | 0.82 (42) | 0.44 (74) |  |  |  |
| Particularism (PA) | 0.80 (42) | 0.70 (41) | 0.75 (35) | 0.60 (41) | 0.58 (38) |  |  |
| Embeddedness (EM) | 0.61 (56) | 0.67 (70) | 0.65 (46) | 0.58 (69) | 0.61 (59) | 0.62 (40) |  |
| Family Ties | 0.65 (52) | 0.56 (70) | 0.70 (39) | 0.50 (68) | 0.42 (67) | 0.49 (32) | 0.74 (52) |
| Significant at 0.001 level unless otherwise noted. | | | | | | | |

# ROBUSTNESS CHECKS

## redundant measures

F&T use two measures of in-group favoritism—strength of family ties and assortative sociality. The second measure is a composite of the strength of family ties measure and a measure based on religiosity. The first and second measure are highly correlated (ρ > 0.95) so we focus on the direct measure of family ties . However, results do not change if the second measure is used. F&T also use two measures of contempory pathogen stress—non-zoonotic pathogen stress and combined pathogen stress, These are also highly correlated (ρ > 0.95), and for clarity we use the more direct measure. Again results do not change if combined pathogen stress is used .

## SENSITIVITY OF EFFECTS to DIFFERENT CONTROL SETS

In Tables 4.S. and 5.S. we examine how the effect sizes and p-values change for key relationships when controlling for: (1) World Region, (2) World Region + measures for the alternative hypothesis, (3) World Region + Dominant religion. After regional controls only one pathogen stress variable, historical pathogen prevalence, remains. After control for government effectiveness of world religion, that measure only remains significantly associated with one measure of in-group investment

| **Table S4. Relationship of 3 major in-group preferences with alternative measures of pathogen stress using three sets of controls** | | | |
| --- | --- | --- | --- |
|  | **Collectivism** | **In-group Favoritism** | **Strength of Family Ties** |
| **Regional control** |  |  |  |
| Non-zoonotic Pathogen Stress | 0.26 | 0.41* | 0.32 |
| Zoonotic Pathogen Stress | 0.01 | 0.10 | -0.03 |
| Historical Pathogen Prevalence | 0.31* | 0.51*** | 0.60*** |
| **Regional + Government Effectiveness Controls** |  |  |  |
| Non-zoonotic Pathogen Stress | -0.01 | 0.05 | 0.14 |
| Zoonotic Pathogen Stress | -0.14 | 0.00 | -0.05 |
| Historical Pathogen Prevalence | 0.06 | 0.17 | 0.46*** |
| **Regional and Religion Control** |  |  |  |
| Non-zoonotic Pathogen Stress | 0.04 | 0.18 | 0.15 |
| Zoonotic Pathogen Stress | -0.10 | 0.04 | -0.04 |
| Historical Pathogen Prevalence | 0.02 | 0.20 | 0.36* |
| p < 0.05, ** p < 0.005, *** p < 0.001 | | | |

We apply the same approach to alternative measures of government effectiveness or material security/insecurity. All measures remain significantly associated with all measures of in-group investment with regional controls or control for non-zoonotic parasite stress. When controlling for religion these measures remain significantly associated with two of the three in-group investment measures.

| **Table S5. Relationship of 3 major in-group preferences with alternative measures of institutions and material security using three sets of controls** | | | |
| --- | --- | --- | --- |
|  | **Collectivism** | **In-group Favoritism** | **Strength of Family Ties** |
| **Regional Controls** |  |  |  |
| Government Effectiveness (1996) | -0.52*** | -0.77*** | -0.38*** |
| Log(GDP 1996) | -0.49*** | -0.80*** | -0.40*** |
| Human Development Index (1995) | -0.51*** | -0.89*** | -0.46*** |
| % Household budget on food | 0.42*** | 0.64*** | 0.28* |
| **Regional + Non-zoonotic pathogen stress Controls** |  |  |  |
| Government Effectiveness (1996) | -0.52*** | -0.76*** | -0.36*** |
| Log(GDP 1996) | -0.46*** | -0.81*** | -0.36*** |
| Human Development Index (1995) | -0.47** | -0.88*** | -0.42*** |
| % Household budget on food | 0.39*** | 0.62*** | 0.24* |
| **Regional + Religion Controls** |  |  |  |
| Government Effectiveness (1996) | -0.29* | -0.63*** | -0.11 |
| Log(GDP 1996) | -0.26* | -0.67*** | -0.13 |
| Human Development Index (1995) | -0.35* | -0.73*** | -0.23 |
| % Household budget on food | 0.26* | 0.51*** | 0.07 |
| p < 0.05, ** p < 0.005, *** p < 0.001 | | | |

## WITHIN REGION ANALYSIS

To examine associations within the major world regions, we conducted regressions for each region predicting each of the 8 measures of in-group favoritism with the following two predictors—effectiveness of government institutions and non-zoonotic pathogen stress. To avoid small sample sizes, we focused on the 17 samples where there were more than 10 countries within a region which had data on a specific measure of in-group favoritism. All eight measures had sufficiently large samples from Europe and Central Asia. Three measures had sufficiently large samples for East Asia and for Latin America and the Caribbean. Two measures had sufficiently large samples for sub-Saharan Africa, and one measure had a sufficiently large sample for Middle East and North Africa. South Asia never had sufficiently large samples.

Among these 17 samples, GE significantly increased in-group favoritism in 12 (8 of 8 in Europe and Central Asia, 2 of 3 East Asia, and 2 of 3 in Latin American and Caribbean). Notably, PS significantly increased in-group favoritism in only 1 sample.

## EFFECT OF 3 In-group preference measures on economic growth, Human Development INdex, and Government Effectiveness

| **Table S6. Standardized coefficients of 13-year change in GDP per capita and quality of public services by three major in-group favoritism measures. Adjusting for region and religion** | | | |
| --- | --- | --- | --- |
| **Variables** | **Collectivism** | **Ingroup Favoritism** | **Strength of Family Ties** |
| GDP_2009_/GDP_1996_ | 0.25* | 0.26** | -0.04 |
| HDI_2010_ –HDI _1995_ | -0.10 | 0.21* | -0.07 |
| PublicService_2009_ –PublicService_1996_ | 0.14 | 0.15 | -0.13 |
| * p < 0.05, ** p < 0.005, *** p < 0.001 | | | |

## INSTRUMENTAL VARIABLE ANALYSIS

Following work in economics , we deploy the mortality rates of early settlers in European colonies (1600-1875) as an instrumental variable which is expected to affect contemporary government effectiveness (see below). Acemoglu et al. provide ample historical evidence that Europeans avoided settling in places with high mortality for Europeans, such as in the Belgian Congo, and instead set up extractive systems in these places. In situations of low mortality, on the other hand, colonizers settled in larger numbers and brought with them institutions, such as respect of private property, checks and balances in government, and equality of opportunity, which in turn fostered greater government effectiveness that persisted even after independence. These measures of settler mortality allow us to identify that portion of the variance in government institutions that is due to early (exogenously caused) settlement patterns. We can then use these predicted values—now unbiased by omitted variables and confounding—to predict our cultural variables and identify a causal relationship. This analysis shows an effect indistinguishable from that observed in the standard regression analysis, which indicates that the standard analysis is unlikely to suffer from the omitted variables biases and confounding that would otherwise jeopardize causal inference.

Using settler mortality as an instrumental variable, we fit a two-stage OLS regression. At level one, settler mortality predicts modern government effectiveness. At level two, the component of government effectiveness predicted by early settler mortality (the predicted value) is used to predict modern in-group favoritism. We include pathogen stress in both regressions to control for any potential confounding due to pathogens in the environment causing both early settler mortality and modern in-group preference (See Table 6.S). Settler mortality was significantly associated with government effectiveness (ρ = -0.54, p < 0.001) and non-zoonotic pathogen stress (ρ = 0.49, p < 0.001).

We use the “robustness check” data series for settler mortality (Acemoglu et al. 2005). We report all results for the sub-samples for which settler mortality is available.

Note that since we are using only former European colonies in this analysis, our sample size is smaller.

A Durbin-Wu-Hausman test assesses whether the estimate from an OLS regression is different from the estimate from instrumental variable regressions. For In-group favoritism and strength of family ties, the estimates from each procedure are not statistically different. Since estimates from instrumental variable (IV) regressions are less efficient than those from OLS regressions, and since there is no difference between the OLS and IV estimates, the OLS regression estimates are preferred. Note, however, that for all three of our dependent measures the IV coefficient estimates are larger in magnitude than the OLS coefficients, and for Collectivism, they are significantly larger in magnitude.

| **Table S7. Comparison of estimated effect of quality of government services on in-group preferences using OLS and instrumental variable estimation (on colonial sub-sample).** | | | |
| --- | --- | --- | --- |
| **Variables** | **Collectivism**  **(n=33)** | **Ingroup Favoritism**  **(n=52)** | **Strength of Family Ties**  **(n=27)** |
| **Panel A: Two Stage Least Squares** | | | |
| Government Effectiveness | -1.95* (1.10) | -0.89* (0.37) | -0.89* (0.42) |
| Non-zoonotic Pathogens | -0.28 (0.35) | -0.06 (0.11) | -0.10 (0.15) |
| **Panel B: First-Stage for Government Effectiveness** | | | |
| Non-zoonotic Pathogens | -0.25 (0.07)*** | -0.20 (0.06)*** | -0.20 (0.09)* |
| Log(settler mortality) | -0.22 (0.13) | -0.28 (0.10)** | -0.38 (0.16)* |
| R^2^ | 0.42 | 0.42 | 0.54 |
| **Panel C: Ordinary Least Squares Regression** | | | |
| Government Effectiveness | -0.49* (0.20) | -0.78*** (0.14) | -0.41* (0.17) |
| Non-zoonotic Pathogens | 0.15 (0.10) | -0.02 (0.06) | 0.06 (0.08) |
|  |  |  |  |
| Durbin-Wu-Hausman Test (p-value) | 0.02 | 0.75 | 0.17 |
|  |  |  |  |
| * p < 0.05, ** p < 0.005, *** p < 0.001. 1-sided tests | | | |

Supplementary Figure 1 plots log(early settler mortality) versus government effectiveness in 1996. Figures 2.S, 3.S. and 4.S. plots settler-mortality-predicted government effectiveness against each of the three in-group preference measures.

**Figure S1.** Government effectiveness (1996) against log(settler mortality).


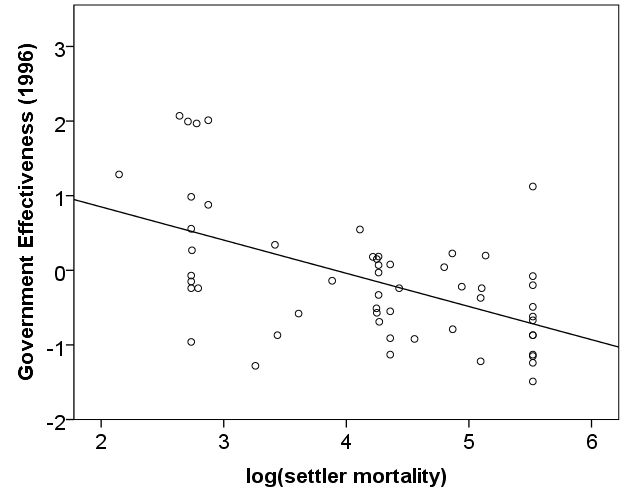


**Figure S2.** Collectivism by Government Effectiveness (as predicted by early settler mortality). Collectivism standardized to mean = 0 and standard deviation = 1.


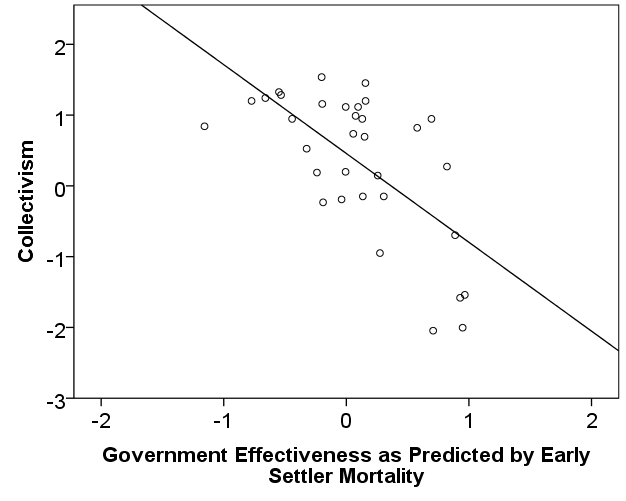


**Figure S3.** In-group Favoritism by Government Effectiveness (as predicted by early settler mortality). In-group favoritism standardized to mean = 0 and standard deviation = 1.


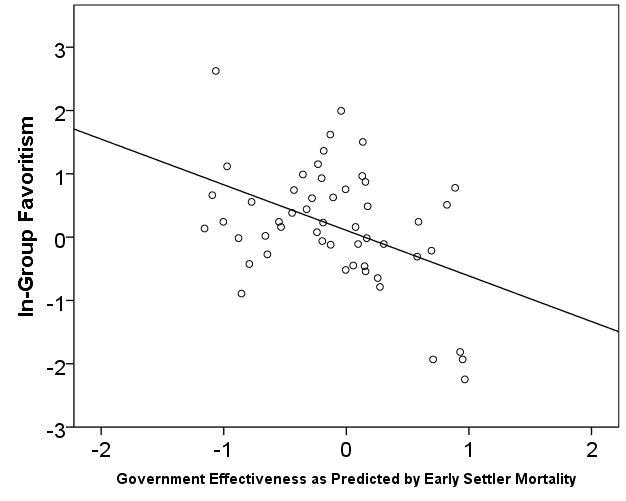


**Figure S4.** Strength of Family Ties by Government Effectiveness (as predicted by early settler mortality). Strength of Family Ties standardized to mean = 0 and standard deviation = 1.


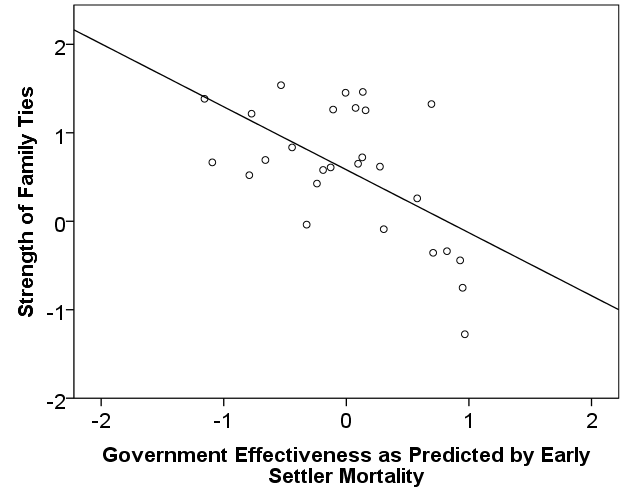


## FULL MODELS for ALL 8 In-GROUP PREFERENCES

Table 8.S. presents the full model (with regional and religion dummies) for all 8 in-group preferences considered in the main text and the supplementary materials. Table 9.S. presents the adjusted R^2^ for the base world region model, and the increase in adjusted R^2^ from adding Government Effectiveness and Pathogen Stress to Model as well as Dominant Religion.

For the simpler model (excluding religion dummies, but keeping regional dummies , non-zoonotic pathogen stress and government effectiveness controls), all eight measures are significantly associated with government effectiveness and none of the eight measures are significantly associated with pathogen stress. We also examined the same model as in 8.S., but including historical pathogen stress instead of contemporary non-zoonotic pathogen stress as a predictor. The relationship with historical pathogen stress was only significant for one of the eight outcome measures (F&T's strength of family ties, p = 0.013).

| **Table S8. Full regressions for each of 8 In-group preference measures by Non-zoonotic Pathogen stress and government effectiveness (with regional and religion controls) :** collectivism (Coll), ingroup favoritism (In), Strength of Family Ties (FT), Cultural Embeddedness (EM), familism (FA), nepotism (NP), compatriotism (CO), particularism (PA). | | | | | | | | |
| --- | --- | --- | --- | --- | --- | --- | --- | --- |
|  | **Coll**  **N = 72** | **In**  **N = 120** | **FT**  **N= 71** | **EM**  **N=71** | **FA**  **N=57** | **NP**  **N=117** | **CO**  **N=76** | **PA**  **N=43** |
| Government Effectiveness | -0.31* | -0.63*** | -0.08 | -0.59*** | -0.62*** | -0.70*** | -0.39* | -0.34 |
| Pathogen Stress | -0.06 | -0.01 | 0.13 | -0.12 | -0.02 | -0.09 | -0.06 | -0.02 |
| **Religion** |  |  |  |  |  |  |  |  |
| Catholic | -- | -- | -- | -- | -- | -- | -- | -- |
| Protestant | -0.27** | -0.33*** | -0.33* | 0.02 | -0.58*** | -0.22** | -0.37* | -0.19 |
| Islam | 0.09 | -0.08 | 0.30* | 0.07 | -0.29* | 0.11 | -0.43* | -0.07 |
| Eastern | -0.03 | -0.08 | -0.08 | -0.04 | -0.25 | 0.01 | -0.22 | -0.17 |
| Orthodox | 0.14 | 0.06 | 0.08 | 0.03 | -0.02 | 0.14* | -0.07 | 0.13 |
| Jewish | 0.02 | -0.09 | -- | -0.11 | -0.22* | -0.01 | -0.39* | -- |
| **Region** |  |  |  |  |  |  |  |  |
| Europe/Central Asia | -- | -- | -- | -- | -- | -- | -- | -- |
| Africa | 0.21* | 0.10 | 0.42*** | 0.45*** | 0.05 | 0.03 | 0.21 | -0.00 |
| East Asia | 0.50*** | 0.17 | 0.26* | 0.48*** | 0.35* | 0.01 | 0.35* | 0.59* |
| South Asia | 0.17 | -0.02 | 0.13 | 0.19* | 0.10 | -0.08 | 0.17 | 0.41* |
| Latin America | 0.49*** | 0.05 | 0.23 | 0.11 | -0.24 | 0.12 | -0.03 | 0.20 |
| Middle East/N. Africa | -0.00 | 0.18* | 0.20 | 0.29** | 0.22* | 0.05 | 0.48*** | -- |
| Adjusted R^2^ | 0.72 | 0.64 | 0.63 | 0.76 | 0.73 | 0.71 | 0.34 | 0.59 |
| ∆R^2^ | 0.03 | 0.22 | 0.00 | 0.16 | 0.22 | 0.30 | 0.06 | 0.01 |
| p < 0.05, ** p < 0.005, *** p < 0.001.  ∆R^2^ = Change in Adjusted R^2^ from adding Government Effectiveness and Pathogen Stress to Model with Regional and Religion dummies. | | | | | | | | |

| **Table S9. Change in Adjusted** R^2^ **by adding Government Effectiveness (GE) and Religion dummies to world region base models** | | | | | | | | |
| --- | --- | --- | --- | --- | --- | --- | --- | --- |
|  | **Coll**  **N = 72** | **In**  **N = 120** | **FT**  **N= 71** | **EM**  **N=71** | **FA**  **N=57** | **NP**  **N=117** | **CO**  **N=76** | **PA**  **N=43** |
| Based Region Model R^2^ | 0.49 | 0.09 | 0.40 | 0.47 | 0.16 | 0.05 | 0.14 | 0.42 |
| GE over Region ∆R^2^ | +0.18 | +0.49 | +0.12 | +0.29 | +0.43 | +0.61 | +0.13 | +0.18 |
| GE over Region & Religion ∆R^2^ | +0.07 | +0.23 | +0.00 | +0.16 | +0.13 | +0.30 | +0.07 | +0.02 |
| Religion over GE & Region ∆R^2^ | +0.05 | +0.07 | +0.11 | +0.00 | +0.15 | +0.05 | +0.08 | +0.00 |

## TESTS OF PROPOSED INTERACTIONS

An earlier reviewer of the paper proposed testing for specific interactions—between pathogen stress and government effectiveness and between government effectiveness and a measure of temperature variability . Here we show the results of those tests, none of which were significant

| **Table S10. Interaction of Government Effectiveness and Non-zoonotic pathogen stress (adjusted for regional controls, standardized beta coefficients):** | | | |
| --- | --- | --- | --- |
|  | **Collectivism** | **In-group Favoritism** | **Strength of Family Ties** |
| Government Effectiveness | -0.49*** | -0.72*** | -0.24 |
| Pathogen Stress | 0.01 | 0.09 | 0.21 |
| Government Effectiveness*Pathogen Stress | 0.04 | 0.05 | 0.13 |
| p < 0.05, ** p < 0.005, *** p < 0.001. | | | |

| **Table S11. Interaction of Government Effectiveness and Temperature Variability (see Van der Vliert 2011) (adjusted for regional controls, standardized beta coefficients):** | | | |
| --- | --- | --- | --- |
|  | **Collectivism** | **In-group Favoritism** | **Strength of Family Ties** |
| Government Effectiveness | -0.46*** | -0.73*** | -0.41*** |
| Temp Range | 0.00 | -0.04 | -0.59*** |
| Government Effectiveness*Temp Range | -0.11 | -0.08 | 0.00 |
| p < 0.05, ** p < 0.005, *** p < 0.001. | | | |

## ASSESSING CONFOUNDING BY HISTORICAL PATHOGEN STRESS

It is possible that the association between institutions and in-group favoritism are confounded by historical pathogens. We address those two concerns here. First, it is possible that historical pathogen stress is an underlying confounder which independently inhibited modern-day institutions and fostered modern tendencies to in-group investment. When we adjust for historical pathogen stress, the relationship between institutions and in-group investment remains significant and maintains a similar strength for in-group favoritism and reduced strength for the other two measures. These findings suggest that this first alternative hypothesis cannot account (at least fully) for the observed effects.

The second hypothesis is that settler mortality led to settlement patterns which independently: (1) inhibited specific institutions and (2) led to inter-group division and hence in-group investment.  This is easily checked by estimating the effect of institutions on in-group investment after controlling for early settler mortality.  When we adjust for early settler mortality the relationship between institutions and in-group investment remains significant for the measures of collectivism and in-group favoritism. These findings suggest this alternative hypothesis is implausible.

| **Table S12. Standardized beta for effect of Government effectiveness on 3 measures of in-group favoritism** | | | |
| --- | --- | --- | --- |
|  | **Collectivism** | **In-group Favoritism** | **Strength of Family Ties** |
| No control | -0.68*** | -0.74*** | -0.52*** |
| Controlling historical pathogens | -0.50*** | -0.66*** | -0.20* |
| Controlling early settler mortality | -0.43* | -0.67*** | -0.40 |
| p < 0.05, ** p < 0.005, *** p < 0.001. | | | |

## MEDIATION Analysis: Government Effectiveness as a MeDIATOR of the effect of Historical PATHOGENS on

To assess the plausibility of the hypothesis that historical pathogens have an effect on contemporary levels of in-group favoritism through the effect of institutions, we conducted a mediation analysis. Historical pathogen prevalence is associated with government effectiveness even after adjusting for regional dummies (standardized beta = -0.51, p < 0.001). To test for mediation of the relationship between historical pathogens and current in-group favoritism by government effectiveness, we conducted a bootstrap mediation test for each of the eight measures of in-group favoritism.

Contemporary government effectiveness significantly mediated the effect of historical pathogen prevalence on seven of the eight in-group preferences (p < 0.05), with the effect on compatriotism marginally significant (p < 0.10).

1. Van de Vliert E (2011) Climato-economic origins of variation in ingroup favoritism. Cross-Cultural Psychology 42: 494-515.

2. House RJ, Hanges PJ, Javidan M, Dorfman PW, Gupta V (2004) Culture, leadership, and organizations: the GLOBE study of 62 societies. Thousand Oaks, CA: Sage.

3. Blanke J, Loades E (2005) The executive opinion survey: an essential tool for measuring country competitiveness. In: Lopez-Claros A, Porter ME, Schwab K, editors. The Global Competitiveness Report 2005-2006. New York: Palgrave MacMillan.

4. Inglehart R, Basanez M, Diez-Medrano J, Halman L, Luijkz R (2004) Human Beliefs and Values: A Cross-Cultural Sourcebook Based on the 1999-2002 Values Survey. Mexico: Siglo XXI Editores.

5. Hofstede GH (2001) Culture's Consequences: Comparing Values, Behaviors, Institutions, and Organizations across Nations. Thousand Oaks, CA: Sage Publications.

6. Triandis HC, Bontempo R, Villareal MJ, Masaaki A, Lucca N (1988) Individualism and collectivism: cross-cultural perspectives on self-ingroup relationships. Journal of Personality and Social Psychology 54: 323-338.

7. Suh E, Diener E, Oishi S, Triandis HC (1998) The shifting basis of life satisfaction judgments acros cultures: emotions versus norms. Journal of Personality and Social Psychology 74: 482-493.

8. Schwartz S (2006) A theory of cultural value orientations: explication and applications. Comparative Sociology 5: 137-182.

9. Parsons T, Shils E (1951) Toward a General Theory of Action. Cambridge, MA: Harvard University Press.

10. Trompenaars F (1998) Riding the Waves of Culture. New York: McGraw Hill.

11. Trompenaars F (1996) Resolving international conflict: culture and business strategy. Business Strategy Review 7: 51-68.

12. Trompenaars F (1993) Riding the Waves of Culture. London: Brealey.

13. Norris P, Inglehart R (2004) Sacred and Secular: Religion and Politics Worldwide. Cambridge: Cambridge University Press.

14. Nations U (1998) Human Development Report 1998: United Nations.

15. Barrett DB, Kurian GT, Johnson TM (2001) World Christian Encyclopedia. Oxford: Oxford University Press.

16. Fincher CL, Thornhill R (2012) Parasite-stress promotes in-group assortative sociality: the cases of strong family ties and heightened religiosity. Behavioral and Brain Sciences 35: 61-79.

17. Acemoglu D, Johnson S, Robinson JA (2001) The colonial origins of comparative development: an empirical investigation. The American Economic Review 91: 1369-1401.

18. Preacher KJ, Hayes AF (2004) SPSS and SAS procedures for estimating indirect effects in simple mediation models. Behavior Research Methods, Instruments, and Computers 36: 717-731.

1. Data accessed from http://info.World Bank.org/governance/wgi/index.asp on November 1, 2011. [↑](#endnote-ref-2)
2. Data accessed from <http://www.fao.org/economic/ess/ess-fs/fs-data/ess-fadata/en/> on November 1, 2011. [↑](#endnote-ref-3)
